# ‘Problems of Today and Tomorrow’: Prevention and the National Health Service in the 1970s

**DOI:** 10.1093/shm/hkz018

**Published:** 2019-02-21

**Authors:** Peder Clark

**Keywords:** prevention, National Health Service, public health, individualism, citizenship

## Abstract

A consensus developed around prevention in the early 1970s as a response to epidemiological studies that had highlighted smoking, diet and physical inactivity as risk factors for chronic disease, especially heart disease. This reaction was catalysed by the financial pressures the National Health Service (NHS) was experiencing, the 1974 reorganisation of the service and international awareness of the Lalonde report. Such widespread interest resulted in three different but contemporaneous reports on prevention in 1976 and 1977. All three emphasised, to varying degrees, personal responsibility and lifestyle as important tenets of prevention. This article focuses on *Prevention and Health: Everybody’s Business*, a 1976 discussion paper published by the four governments of the UK, to explore this preoccupation with disease prevention throughout the decade, and what it reveals about public health in Britain, political attitudes to the NHS and the changing relationship between citizenship and the welfare state.

In Spring 1976, the four governments of the UK published *Prevention and Health: Everybody’s Business*, a discussion paper that outlined ‘a reassessment of public and personal health’. It argued that one potential solution to the financial problems then facing the National Health Service (NHS) was a shift from a curative service to one that promoted health instead. Prevention, it reckoned, was better than cure, not least for the NHS’ bottom line. Furthermore, in facing what the report described as ‘the problems of today and tomorrow’, it was necessary for everybody to take ‘personal responsibility’. Stating that ‘[w]e as a society are becoming increasingly aware of how much depends on the attitude and actions of the individual about his health’, it drew attention to some of ‘those diseases the cause of which and the solution to which can be laid at the door of man’s behaviour’.^1^ Citing ‘smoking-related diseases, alcoholism and other drug dependencies, obesity and its consequences, and the sexually transmitted diseases’, it concluded that ‘it is clear that the weight of responsibility … lies on the shoulders of the individual himself’.^2^

*Prevention and Health* was just one of a profusion of publications and reports on disease prevention during the 1970s. Concurrent to *Prevention and health* was a report produced by a joint working group of the Royal College of Physicians (RCP) and the British Cardiac Society (BCS) entitled *Prevention of Coronary Heart Disease* and an inquiry into ‘preventive medicine’ by the Social Services and Employment Subcommittee of the Expenditure Committee of the House of Commons.^3^ A White (‘command’) paper was produced by the government in response to the latter inquiry, and booklets on a variety of topics were produced as tributaries to the main *Prevention and Health* publication.^4^

This article uses *Prevention and Health* to explore this preoccupation with prevention, and what it reveals about public health in Britain, political attitudes to the NHS in the 1970s and the changing relationship between citizenship and the welfare state. While the idea of prevention was hardly new, it acquired a fresh political urgency during this decade. Virginia Berridge notes that:

[b]y the 1970s, a new style of public health was emergent, both nationally within the UK and internationally as well … [which] stressed the role of individual prevention and responsibility for health, with its roots in the earlier 1950s epidemiological “paradigm shift” epitomized by smoking and lung cancer. The concept of the “risk-avoiding individual” replaced the mass vaccination campaign image of 1950s public health.^5^

*Prevention and Health* marked the juncture at which lifestyle—a focus on smoking, drinking, diet and exercise—became codified in public health policy. It drew public attention to non-communicable diseases, particularly coronary heart disease, as the predominant causes of morbidity and mortality in the UK. It presented the responsibility for the prevention of these diseases almost entirely within the ambit of the public themselves. Indeed, Charles Webster suggests that ‘[i]n essence, the sick were accused of bringing ill health upon themselves and thereby wasting the resources of the NHS.’^6^ By the quantities in which they ate, drank, smoked and exercised, the public would be responsible for preventing disease; or conversely, bringing it upon themselves.

At the same time as the emergence of the ‘risk-avoiding individual’, governments across the world, and particularly in Britain, were dealing with a series of challenges to the status quo of the welfare state, from fiscal squeezes brought about by oil crises to ideological attacks from the New Right, or what Geoff Eley describes as the ‘long and painful dismantling’ of the post-war settlement.^7^  *Prevention and Health* explicitly addressed the first of these, with its optimistic belief that prevention might hold the key to NHS financial shortfalls. The latter critiques were more implicitly acknowledged, revealed by health minister David Owen’s comment to *The Times* immediately prior to publication that ‘a basic rethink of a lot of different attitudes concerning health service provision’ was needed.^8^

This conflation between the focus on the individual’s role in preventing ill health and the challenges facing the health service was not only at the heart of *Prevention and Health*, but it also provides a lens with which to view a broader picture of the citizen and their relationship to the welfare state. Because prevention was ‘everybody’s business’, no citizen was excused from adapting or changing their lifestyle accordingly. Personal responsibility was emphasised, and even, as discussions of the draft will reveal, was considered as part of the social contract for a health service free at the point of use. Indeed, the actions of the individual in attending to aspects of diet, consumption and exercise considered healthy were part of what Matthew Grant describes as ‘active citizenship’.^9^  *Prevention and Health’*s focus on personal responsibility also provides an example of an appeal to what recent scholarship identifies as the ‘popular individualism’ of the 1970s.^10^ The moment of crisis that this decade has come to represent was also a time of opportunity, in which ‘a diverse “marketplace of ideas” could flourish’.^11^ This attempt at fresh thinking was apparent in *Prevention and Health*, with its eyes trained on ‘today and tomorrow’ and its ideas about ‘action which individuals take in relation to the health and well being of themselves and their family’.^12^

The tensions between these two concepts illustrate the contradictions at the heart of *Prevention and Health* during this pivotal decade for the welfare state. While ‘popular individualism’, by its very nature, largely describes a bottom-up concept, *Prevention and Health* suggests that policymakers and politicians were starting to conceive of the British public in this way, ahead of its apparent apex in the 1980s. For Emily Robinson and her colleagues, individualism was not ‘the result of Thatcher … If anything, it was a cause of Thatcherism’.^13^ Personal responsibility and such individualism were elided by *Prevention and Health*. The type of citizen that the document’s authors had in mind was a somewhat classless, self-sufficient individual who nonetheless paid attention to and participated in debates about social and political issues, such as the budgetary pressures on the NHS or the rising tide of non-communicable diseases. Surprisingly, Grant’s ‘active citizenship’ neglects to mention health, but this useful conception, in tandem with his historicisation of ‘legal’ and ‘formal citizenship’, sheds light on the preventative agenda’s expectations of the ‘good’ citizen, and their role in ensuring that the NHS was still a viable pillar of the welfare state. Finally, and paying due attention to Grant’s insistence that citizenship is ‘a concept with historically and culturally specific meanings’, it is also vital to place prevention in the context of discourse around the NHS going into the 1970s.^14^ Alongside its first major reorganisation in 1974, fundamental questions were being asked of the NHS, as illustrated by a widely viewed television programme marking its twentieth anniversary in 1968, which punningly and provocatively posed the question of whether the British public were getting *Something for Nothing.*^15^ While, as shall be demonstrated, debates around preventive health had international salience, it was in the political and social context of Britain that they gained particular traction.

In exploring these issues, the article will first detail the medical, political and policymaking consensus that was built up around prevention in the early 1970s, before examining the construction of a discourse around personal responsibility in *Prevention and Health* itself and, finally, assessing the success or otherwise of the preventative agenda in engaging the public and achieving Owen’s ‘basic rethink’.

## ‘Lip Service’: Creating a Consensus Around Prevention, 1971–75

In delivering a speech to the Royal Society of Health’s Congress of 1971, the Chief Medical Officer (CMO) Sir George Godber gave his thoughts on what he viewed as the key challenges for preventative medicine for the rest of the decade.^16^ In looking forward, he was also obliged to glance back, noting the longer history of prevention. Godber’s speech marked crossroads, heralding the efforts of Victorian social reformers such as Edwin Chadwick and former CMO John Simon in preventing infectious diseases, while acknowledging that the public health service was facing new challenges, particularly with non-communicable diseases. For Godber,

preventive medicine must somehow convey its message more effectively to the general public … health education is most needed to persuade people to do or refrain from doing things for themselves for their long term benefit.^17^

If Godber’s overview had included more recent history, he might have noted his predecessor George Newman’s treatise on ‘Preventive Medicine’ or the the interwar efforts of the Central Council for Health Education from 1927 with its ‘ideals of morality and citizenship’.^18^ But nonetheless, Godber’s thoughts were reflective of broader ‘signs of a change of direction for health education’, signalled by the Cohen report in 1962, and the formation of the Health Education Council in 1968.^19^

In 1972, leading epidemiologist Jerry Morris gave his own take on Godber’s theme in an address at the Royal Society of Medicine. Morris was an influential figure in post-war public health, whose apparent shift of interest from structural determinants of health to the behaviour of individuals was, according to Dorothy Porter, reflective of wider trends in the field.^20^ Supplementing the CMO’s narrower focus, Morris spoke of ‘four principles of attack, four strategies’ to prevention in ‘a society like ours, its health problems dominated by the “chronic diseases”’.^21^ These were: ‘The Quality of Medical Care’; ‘Early Diagnosis’; ‘Protecting the Vulnerable Individual’ and, finally, ‘A Healthier Mode of Life’. Referring to the ongoing forceful public debates on cholesterol—‘[o]ne of the malnutritions of affluence is the rising consumption particularly of dairy fat’—and acknowledging that manufacturers and industry had a part to play, Morris argued that to ‘influence the social pattern and prevalent lifestyles and shift norms of behaviour … is far the best way’ to address poor diet and low exercise levels. Morris concluded, therefore, that ‘[p]revention today is often a matter of individual and family behaviour in a society too often exerting the wrong pressures.’^22^

Later in the decade, Tom Meade, the director of the Medical Research Council’s Epidemiology and Medical Care Unit, penned an article in *The Lancet*, which encapsulated the abiding concerns of the preventative agenda in the 1970s. Meade developed the themes of Godber and Morris, not only emphasising the need for better communication and a focus on chronic diseases but also folding in a third, particularly politically important aspect: the economic imperative to prevent. Observing that in the wake of the 1973 OPEC oil crisis and the ensuing global recession, ‘resources have become increasingly stretched over the past few years’ and that ‘[l]ike virtually all health-care systems, the NHS is geared predominantly to a policy of managing established disease’, Meade argued it was ‘quite simply, coping with the wreckage of our failures to prevent.’ Drawing on Archie Cochrane’s 1972 pamphlet *Effectiveness and Efficiency*, Meade noted that while ‘the central dilemma for health care at the present time’ was not new, he thought its ‘intensity’ was throwing out a challenge: ‘are we serious about prevention? Are we serious about “trying to turn off the tap”?’^23^

Indeed, given the economic climate, and the solution that it might offer to the problem of ever more finite resources, discussions around prevention could hardly remain limited to public health circles. Historian Rudolf Klein suggests that the ‘rhetoric of financial crisis rose to a crescendo in the second half of the 1970s’ and provided ‘background music’ to much of the public and political discourse around the NHS during this period.^24^ In 1978, the Office of Health Economics summed up the key features of this debate. Firstly, that ‘even when adjusted for the falling value of the pound … the NHS costs three times as much as when it was first established’. Secondly, that this increase was particularly acute during this decade and not matched by macroeconomic performance; NHS costs had ‘risen by 39 per cent since 1970, as compared to a 13 per cent growth in Gross National Product’.^25^ As early as 1972, the Conservative Political Centre (CPC) published a pamphlet by Trevor Weston, a GP known for his occasional appearances on *Women’s Hour*, which made an argument that would be wearily familiar by decade’s close: that in an increasingly stretched NHS, more focus was needed on personal, preventative health initiatives, such as health education, early diagnosis and screening.^26^

But it was the report penned under the name of the Canadian Minister for Health and Welfare Marc Lalonde, entitled *A New Perspective on the Health of Canadians*, which would provide the impetus for prevention to be seriously investigated in British political and policy circles.^27^ Published as a working paper, the Lalonde report introduced the conceptual framework of the ‘health field’, in which ‘human biology, lifestyle, environment and health care organization’ were the four components that influenced health and disease.^28^ Drawing on contemporaneous critiques by British epidemiologist and medical historian Thomas McKeown, it ‘shattered the conventional belief that healthcare services were the foundation for future improvements in population health’.^29^  *A New Perspective* quoted McKeown’s contention, based on historical demographic data, that ‘[p]ast improvement has been due mainly to modification of behaviour and changes in the environment and it is to these same influences that we must look particularly for further advance.’^30^ McKeown’s controversial argument, that improvements in life expectancy were attributable to factors other than medical care, was heavily contested throughout the late 1960s and early 1970s but proved highly influential for advocates of prevention.

The Lalonde report also suggested that ‘high-risk populations’, such as ‘candidates for coronaries’, could be identified; ‘an obese man who gets little or no exercise, ingests excessive amounts of animal fats, smokes cigarettes, drinks a lot of coffee and works in a high pressure job’ was one such example.^31^ Using this analysis, *A New Perspective* proposed, alongside complementary regulatory, research, healthcare efficiency and goal-setting strategies, the development of a health promotion strategy, ‘aimed at informing, influencing and assisting both individuals and organizations so that they will accept more responsibility and be more active in matters affecting mental and physical health’.^32^

Godber, writing in 1977, articulated the prevailing contemporary judgement on the Lalonde report, stating that it ‘had a worldwide effect in making governments as well as the health professions realize that the promotion of health in future depends more on the pattern of living adopted by the individual than on technical or allied procedures’.^33^ However, beneath this apparent clarity of purpose, political theorists have identified the use of the document by different factions that reveal more about the reader than the text. Theodore R. Marmor and Albert Weale have suggested that ‘its diverse, and sometimes inconsistent, messages can be picked out and amplified by various self-interested groups to their own advantage’.^34^ For example, Robert Evans has noted that the private companies could seize upon the lifestyle aspects for marketing purposes, federal and government bodies could use it to justify increased control over healthcare costs, while more radical critiques suggested the way in which the lifestyle message was adopted meant that structural determinants of health were neglected.^35^

Indeed, perhaps the principle of prevention, which few could argue with, provided cover for a diverse range of interests throughout the 1970s in the UK. Likewise, both the novel status of the Lalonde report and its apparent pliability made it attractive to policymakers across the world. An editorial in *The Lancet* praised *A New Perspective* as a ‘radical rethink’ after ‘no end of lip-service to the cause of prevention’, concluding that ‘[o]thers outside Canada will certainly profit by listening; perhaps they can also join in.’^36^

In England, the recently promoted Minister of State for Health and Social Security David Owen was keen to do just that. A rare example of a clinically trained health minister, but more importantly both a forceful personality and political opportunist, Owen read *A New Perspective* in the summer of 1974.^37^ Following Labour’s October election manifesto pledge to ‘put the emphasis on prevention and primary care’, Owen asked the Department of Health and Social Security (DHSS) to produce its own policy discussion document on preventative health.^38^

Work began in earnest in February 1975. A retired epidemiologist and former Principal Medical Officer in DHSS, G. Wynne Griffith was commissioned to write a draft, with a steering committee chaired by the deputy CMO John Reid to review the field and steer the document towards publication. It was agreed that representatives from Scotland, Wales and Northern Ireland health departments would ensure ‘a full United Kingdom approach to the subject’.^39^ Like *A New Perspective*, ‘it was not to be a statement of Government policy; it was to be a consultative document’.^40^ At this early stage, its ambitions were modest. It was intended to provoke discussion but not too much. Clearly with one eye on the excitement that the Lalonde report had produced, the steering group noted that

[i]t would be extremely important to avoid the appearance of announcing a brave new era of prevention, and thereby creating irresistible public pressures; the consultative process would need to be carefully controlled, lest it generate impetus in unproductive directions.^41^

The early discussions of the committee centred on economic imperatives and general principles, but in medical circles, much of the imperative for prevention was concerned with the nation’s major killer, coronary heart disease. It had been frequently used as a prime example by general prevention pieces, such as *A New Perspective* or Morris’ ‘Four Cheers for Prevention’, and a spate of research articles and opinion pieces continued to appear in the medical press. One of the more influential was penned in 1973 by cardiologists Richard Turner and Keith Ball and promised ‘a counter-blast to present inactivity’. Claiming that ‘[c]riticism and apathy concerning the prevention of coronary heart-disease (C.H.D.) is often based on ignorance of what has already been established’, it vigorously argued that ‘[s]ince complete proof may never be forthcoming, action should be taken now on the basis of strong probability.’^42^ After running through the most common risk factors (diet, smoking, physical inactivity, stress) and noting that they were ‘not only individually adverse, but cumulative’, Turner and Ball proposed that screening for disease in ‘symptom-free individuals at high risk’ via ‘regular health examinations’ was the only way to prevent ‘[p]otentially the greatest epidemic man has faced.’^43^ Screening for cervical cancer via the smear test had been introduced by the NHS in 1967, while debates on screening for other conditions had centred on technical issues such as the sensitivity and specificity of any proposed tests and the availability of resources.^44^ Turner and Ball’s proposal suggested that following initial screening, the physician could then provide tailored lifestyle advice to the individual. The authors shrugged off the resource implications of such a comprehensive programme by insisting that

The cost would be small compared with the saving which would result … in Britain little more than lip-service is being paid to prevention … Far larger sums are being spent on the provision of acute coronary care and facilities for myocardial re-vascularisation than on tackling the problem of preventing the condition ever occurring.^45^

Ball and Turner proposed a personalised lifestyle approach, using evidence collected from epidemiological studies to calculate the risk of future disease for an individual based on their behaviours. An editorial in the Lancet the following year, however, revealed some of the contradictions inherent in this type of programme. Whilst acknowledging the weight of epidemiological evidence that accumulated over the last 20 or so years, it outlined the pitfalls of applying this at an individual level:

All that is known from epidemiology and group studies which depend on statistical expression of mean differences should be emphasised as representing just that, a mean difference between compared groups. Identification of a susceptible individual has never really been achieved except through a gross averaged assessment of accumulated risk factors … a prediction within a high risk group on the basis of multiple factors still produces incorrect forecasts more often than correct ones.^46^

Despite this rare exposé of the ‘black box’ of risk factor epidemiology, *The Lancet* stopped short of condemning an approach that focussed on the individual.^47^ While the journal’s editorial writers believed the implementation of a screening programme would not be merited, ‘we should accept the hotchpotch of hard evidence, suggestion, and faith as our guideline’. Individuals should be given lifestyle advice that was ‘reasonable as well as objective’, and that in some areas, such instruction could be given with more conviction: ‘Smoking is a hazard to health and can be positively discouraged’.^48^ The two articles had contrasting views on the credibility of epidemiological evidence but shared a belief in firstly the intrinsic value of lifestyle advice and secondly, a largely unstated assumption that if such suggestions were delivered by a trustworthy source, such as a medical professional, they would be acted upon by members of the public. Such suppositions would underpin much of the political and policy debate around prevention.

Both Ball and Turner were parties to an announcement that would further draw the DHSS working group’s attention to heart disease. They were members of a joint working party convened between the BCS and RCP that announced its intention to publish a report on the prevention of coronary heart disease at the same time as the proposed DHSS document in early 1976. This concerned some members of the DHSS group, who worried that ‘definitive publications [that] might emanate from different bodies’ would compete with their own publication. One solution to this problem of primacy was that it be ‘printed and circulated in the Health and Social Subjects series … if there was a definite offer to do this I think that it would be accepted’.^49^ Such an invitation was never made, but this example illustrates, on the one hand, the large degree of consensus on principles between the different groups, and, on the other, the sense of competition that was felt, a consequence of so much overlapping activity on prevention.

Meanwhile, the drafting of the DHSS document continued apace. These drafts, and comments on the work-in-progress from civil servants, reveal the different conceptions of prevention being explored. Griffith defined preventative medicine ‘as the application of knowledge with the aim of preventing disease, disability, injury or premature death’. Griffith argued that this was ‘everybody’s business: not only doctors and not only health professionals but the individual member of the public too’.^50^ Commentators were largely in agreement with this statement but felt that it might go further too: ‘Since what is everyman’s business is often no one’s responsibility, could responsibility be emphasised?’^51^

If prevention was an individual responsibility, it was also a citizenly duty. Increasing demand for the NHS was a driving force for the fashion for prevention, particularly from those on the right of politics, as illustrated by the CPC’s pamphlet. Aware that it might be politically contentious, nonetheless members of the working party felt it merited exploring:

I have in mind here a discussion of the attitudes and degree of responsibility it is reasonable to expect from the good citizen as a quid pro quo for a ‘free’ comprehensive Health Service. It brings in such matters as safety on the roads and in sport, drink and driving, family planning as well as some angles on such things as obesity and smoking … This may be a difficult subject, but I should have thought worth tackling.^52^

The ongoing financial constraints in the NHS added a further dimension, with Griffith arguing that a symbiotic relationship existed between healthcare use and prevention:

Public education should be directed both at inculcating healthy ways of living and at more intelligent use of services; in that way it should be possible to release resources for prevention.^53^

More sceptical civil servants disagreed, scoffing that

any claim that greater emphasis on preventive medicine would enable us to contain the cost the NHS would be found … to be as fallacious as the Beveridge theory that the demand for Health Services would flatten out as the nation’s health was improved by the Service.^54^

Similarly optimistically, Reid also saw the 1974 reorganisation of the NHS as a good opportunity, believing that ‘the time is now ripe for a gradual reorientation of the Health Service towards prevention’.^55^ The restructure had moved the public health service from local government into the NHS, but it was not this development that he had in mind. Rather, the creation of Community Health Councils (CHCs), intended to increase patient voice and public participation in NHS decision-making, were seen by Reid as ‘a valuable potential opportunity for involving the public in prevention’.^56^ Without such involvement, Reid worried that ‘prevention will continue to earn little more than lip service’.^57^

This anxiety about ‘lip service’ extended to discussions about the intended audience of the document and was amplified by concerns with Griffith’s somewhat ponderous style. One civil servant described it as ‘yet another text book’ with a ‘slightly idiosyncratic point of view’, while another complained that ‘it is difficult to decide at whom it is directed … It contains the sort of material one tends to find in articles of the “Whither Medicine?” type which appear in medical journals on centenary occasions’.^58^ Beyond the lack of punch in the prose, the working group found it hard to imagine how the document could foster discussion among the public, as they warned Reid to ‘watch that the final results … really highlight the consultative nature of the document and the areas in which response is particularly required’.^59^

Different models of prevention had been proposed, from individual screening to the involvement of CHCs, and different motivations drove various actors, from cost containment to halting the coronary heart disease epidemic. What most could agree upon, however, was that preventative efforts required the public’s participation, with individuals taking responsibility for their own health. Enough ‘lip service’ had been paid to the concept; action was needed.

## ‘Personal Responsibility’: Lifestyle as Preventive Medicine, 1975–77

Outside of Alexander Fleming House, word was getting around that the DHSS was planning to publish a document on prevention. Announcing its publication for the following year in response to a parliamentary question in June 1975, Owen stated that ‘for both humanitarian and economic reasons the Government are anxious that where practicable greater priority should be given to the development of preventive services’.^60^ A further question, from Janet Fookes MP 2 months later, demanded ‘a high-level inquiry into the possibility of extending the scope of preventive medicine’, stating that ‘we have in the past paid too little attention to the old adage about prevention being better than cure’. Owen agreed that ‘we pay too much lip service … if there is any responsibility for the lack of action on preventive medicine it probably lies in this House’.^61^

Evidently, MPs on both sides of the House saw this as a challenge. Within a couple of months, and apparently prompted by Owen’s statement, a parliamentary inquiry had been initiated on preventative medicine, led by the Social Services and Employment Subcommittee of the Expenditure Committee. This meant that there were now three separate reports on prevention being compiled simultaneously: the subcommittee’s inquiry, the DHSS discussion document and the BCS/RCP report on heart disease.

If in retrospect all this activity appears to be a duplication of effort, similar thoughts were voiced at the time.^62^ Nonetheless, the process by which the House of Commons subcommittee reached their conclusions was distinct. As a parliamentary inquiry, views were actively sought from the health professions, experts and academics, industry and the public themselves. The subcommittee held 23 oral evidence sessions and included over a hundred pieces of written evidence in their deliberations. This evidence provides a broader perspective on views of prevention than that in the drafting of the DHSS discussion document, which may only reflect those of policymaking elites. Although memoranda were received from organisations as diverse as the Scottish Whisky Association and the Family Planning Association, this discussion concentrates on evidence pertaining to heart disease and its risk factors, such as smoking, diet and exercise.

An eight-strong delegation from the British Medical Association (BMA) provided oral evidence in May 1976. The memorandum submitted before this appearance was a curious mix of futuristic and reactionary rhetoric. On the one hand, it referenced Alvin Toffler’s *Future Shock* to support the BMA’s assertion that ‘the pace of change within the human race is undoubtedly accelerating at a faster and faster rate’ and that consequently individuals had to ‘make more adjustments throughout his life either than his father or still more his grandfather’.^63^ It railed against the pernicious influence of corporate interests, and in particular the tobacco industry, in distorting public health priorities. But a few paragraphs later, however, the BMA were suggesting that despite this environment, ‘education should be directed … towards increasing the individual’s own sense of responsibility for health’. It followed this with a quite astonishing section, suggesting that unhealthy persons might be made to feel more than mere responsibility:

There is at present no stigma attaching to an admission of medically confirmed ill health, although such ill health may be nothing more than a disguised manifestation of inadequacy. No one willingly admits that they have failed to achieve their social ambitions or to be successful in their career. If ill-health were to be regarded in the same light as social or economic inadequacy it could no longer be excused as being an unavoidable consequence of external and uncontrollable events.^64^

The extent to which such views were shared by other representatives of the medical profession is not altogether clear. The RCP, concentrated their evidence on the then hot topic of fluoridation and dental decay, while the Society of Community Medicine, a small body representing some thousand community physicians, took a diametrically opposed view to the BMA, arguing that ‘educational campaigns fail to persuade more than a minority of individuals to alter their way of life’.^65^

Oddly, Alexander ‘Sandy’ Macara, a public health lecturer who would go on to be chair of council for the BMA in the 1990s, gave evidence on behalf of both organisations. While this may reveal no more than the conflict of interests of one man, it also reflected the prevailing tensions in medical and public health circles. A conviction that the individual was largely responsible for his or her own ill health was countered by a scepticism that health education messages could compensate for the wider influences at play in post-industrial societies. Macara expressed this by suggesting that ‘the whole trend in behavioural patterns in society in the last ten or twenty years has been antagonistic to the sort of message which we should wish to convey’.^66^

This assessment was echoed by some of the experts and academics that the inquiry called upon. Controversial nutritionist John Yudkin, while giving evidence primarily on his particular interest in sugar and its putative link to heart disease, noted that ‘experience with cigarette smoking has demonstrated that giving people information does not necessarily produce changes in behaviour’.^67^ John Butterfield, a leading authority on diabetes, disagreed with most of Yudkin’s evidence but shared his views on health education and its limited role in affecting individual behaviour.^68^ Representatives from the London School of Hygiene and Tropical Medicine (LSHTM), while condemning much health education as ‘conventional propaganda from governmental sources’, gave a qualified endorsement of ‘major community developments with emphasis on group action and mutual support, if we are to alter prevailing norms of behaviour’.^69^

What of the ‘very powerful commercial interests’ that had been identified by the BMA as militating against effective health education campaigns?^70^ While many witnesses who worked in public health had criticised elements of the food and tobacco industries, evidence provided by such corporate interests demonstrated a more complex relationship between the two factions. For example, the Tobacco Research Council revealed that they continued to provide funding for a number of epidemiological studies, including the Whitehall study of civil servants led by Donald Reid, who had provided evidence to the inquiry on behalf of LSHTM.^71^ Corporations were also keen to use epidemiological data to bolster their own arguments regarding prevention, especially when it might dovetail with the promotion of their products. The manufacturers of Flora margarine, van den Berghs and Juergens Limited, repeatedly lobbied the inquiry and ministers more widely to endorse polyunsaturated fats, as well as noting that Jerry Morris, in giving evidence, had mentioned their product by name.^72^ Other food industry bodies, such as the National Dairy Council, highlighted uncertainty in the research literature to try to prevent the committee forming adverse opinions about dairy produce and its potential link to coronary heart disease.^73^

Interest from the wider public was, however, limited. While one correspondent was ‘extremely concerned’ about the ‘possibility of introducing measures to further control the freedom of the individual in his drinking, smoking and physical habits’, there is little evidence that many of his ‘fellow citizens’ joined his petition for the Committee to ‘give us back our England’.^74^

What all this evidence reveals is the cleavages between the DHSS view on prevention and the wider discourse. There was widespread agreement that prevention should be pursued, both as a general principle for a hard-pressed health service and as the means under which any number of interests could pursue their own ends. There was also consensus that the lifestyle of individuals needed to be changed as a crucial element of the preventative agenda, but how this would be achieved was open to interpretation. While DHSS officials were keen on health education, a small but significant tranche of experts already saw it as a busted flush that would only make a difference to a few individuals. Advising that it was better for the public’s health to not smoke, drink less, eat healthily or exercise more was a long way from people actually taking action. Indeed, at a meeting of the Chief Scientists Research Committee, Morris had asked whether the discussion document ‘should not be more interested in behaviour change rather than education … [and] that this involved many matters which are not the prerogative of health departments, for example taxation, advertising and so on’.^75^

While the House of Commons inquiry continued its deliberations, both the DHSS and the BCS/RCP working groups published their missives. *Prevention and Health: Everybody’s Business* was the first out, published on 16 March 1976. Over the course of a couple of years’ gestation, it had gone through a number of iterations, not the least of which was a rewrite by a professional journalist. The presentation of the document was considered as important as the content in attracting the public’s attention: ‘it should have an eye catching and glossy cover (but not appear to be extravagantly printed)’.^76^ Even the colour of the cover merited debate, with Owen personally suggesting that it be red to distinguish it from the usual ‘green’ consultative paper and so ‘blur its status’.^77^ The booklet was sold through government bookshops to the general public, and even its retail price had been considered; ‘a priced document will attract more interest and prestige and … would be more widely available than a Departmental document’.^78^ However, hopes for widespread media coverage were scuppered by wider political events, with Harold Wilson announcing his resignation as Prime Minister on the same day.^79^

As for its message, what would playfully become known as the ‘red book’ offered a brief history lesson, outlining the successes of the prevention and near eradication of much infectious disease, before turning to ‘[s]ome problems of today and tomorrow.’ Noting the major causes of mortality to be ‘heart disease, cancer and stroke, in that order’, it warned that ‘affluence is not an unqualified boon … it has opened the door to [diseases] arising, for instance, from unwise behaviour and over-indulgence in one form or another’.^80^ It outlined two contrasting philosophies (‘Some would put the emphasis on the role of the individual … others would say that the Government should impose more control or do more to educate and persuade the public’)^81^ before making clear which side *Prevention and Health* came down on: ‘today, prevention depends on the attitude of the individual to his own lifestyle’.^82^

The reasons why the document was so strong on personal responsibility are worth disentangling. Perhaps partly it can be explained by the desire to produce a document that would be ‘reasonably controversial in order to enlist the attention of outside readers in the subject of prevention generally’.^83^ While this is plausible, the extent to which the document was steered by the views of Owen is apparent from his personal reflections in *In Sickness and In Health: The Politics of Medicine*, published in the same year:

[*Prevention and Health*] has the object of changing public attitudes so that the National Health Service is not seen as the sole provider of health in this country … We live in a free society where people can do what they like to themselves, but individuals cannot abdicate from their responsibility for their own health … The public puts considerable pressure on doctors to provide health to order while consciously abusing its own health.^84^

Owen’s views were arguably reflective of the wider medical profession as evidenced by the BMA’s memorandum to the parliamentary inquiry. A note by the civil servants drafting *Prevention and Health* also suggests that they were conscious of a broader consensus on the individual’s role in prevention:

The general philosophy of personal responsibility will be attacked by those who believe that the fault always lies with the “system” or by those who would blame their genes for everything … But I would remind you that other commentators have suggested we emphasise the personal responsibility angle even more than we do.^85^

*Prevention of Coronary Heart Disease* emerged a fortnight later. The BCS/RCP joint working group largely held to what had become the orthodox position on prevention. Noting the ‘multifactorial concept of risk’, it argued that there was ‘considerable evidence that the causes of CHD … are rooted in the modern, affluent way of life’.^86^ Consequently, a ‘comprehensive public and professional educational programme will be needed, together with the involvement and co-operation of food manufacturers, educational authorities and the mass media’.^87^ If these positions seemed to chime almost perfectly with those of *Prevention and Health*, such suspicions were confirmed by Gerry Shaper, the chair of the working party, telling *The Times* that ‘[p]revention is now everybody’s business’.^88^ Furthermore, the report was circulated to all doctors, with a covering note by the CMO, drawing its recommendations to their attention.

Both *Prevention and Health* and *Prevention of Coronary Heart Disease* placed personal responsibility and lifestyle at the heart of their messaging. The importance of engaging the public, and educating them to smoke less, exercise more and eat more healthily, was strongly emphasised. But although this consensus had been reached, it had yet to be effectively communicated. The following section explores the responses to *Prevention and Health* and argues that it was this with the public that that the limits of the preventive agenda was reached.

## ‘A Publicity Exercise’: Engaging the Public, 1977–78

The DHSS had intended to start a conversation about prevention and to that end organised a symposium to discuss *Prevention and Health* held at Imperial College London in July 1976. Unsurprisingly, the tone was largely consensual, with Owen giving the opening address and Norman Hale from DHSS presenting evidence that he claimed meant the document was ‘starting to achieve its initial object of re-kindling a nationwide interest in prevention’.^89^ Despite its poor initial press coverage, the DHSS had been broadly pleased with the response to *Prevention and Health*, noting that many of the professional bodies, including the BMA, Royal College of General Practitioners, Health Education Council and Faculty of Community Medicine, had given it ‘an enthusiastic reception’.^90^ Morris, however, sounded a rare note of dissent, noting that

‘Today’s emphasis on personal responsibility for health, on the need for the individual to alter his style of life, is right and necessary and represents a real and major shift. But it is only half the story, and I wonder if we are getting the balance right’.^91^

Others were starting to ask the same question. The House of Commons subcommittee had completed its inquiry in April 1977 and published what the *British Medical Journal (BMJ)* described as ‘a concise and uncompromising report’.^92^ Determined to tell the other side of the story, it recommended much more government action. The report made 58 recommendations, many of which would prove to be prescient of later developments in public health policy, ranging from suggestions that nutritional information be included on food labelling to a call for a ban on smoking in public places. Very few concerned the behaviour of individuals, 12 concerned health education and the majority concerned administrative, legislative or fiscal steps that the government should take. The report was also much taken with the issue of finance, perhaps reflective of the publication the year before of the deliberations of the Resource Allocation Working Party (RAWP), which focussed on how secondary care might be more equitably funded.^93^ While *Prevention and Health* had vaguely suggested that prevention might help with the parlous finances of the health service, the inquiry’s report recommended cutting high technology medicine to pay for, as one example, health education on heart disease.^94^

As a parliamentary inquiry, the government was obliged to react in the form of a White paper, for which ministerial responsibility had switched to the new Secretary of State, David Ennals. Civil servants checked off each of the recommendations, recording whether the DHSS accepted each one or not. But beyond this painstaking exercise, they also wished to defend the stance adopted in *Prevention and Health*. It is at this point that some of the contradictions of the preventative consensus built up throughout the 1970s became most apparent. Civil servants had fretted about how without further government action, and the partnership of the public in prevention, *Prevention and Health* would amount to little more than ‘a publicity exercise’.^95^ The model of prevention that had been built since the beginning of the decade had been predicated upon the lifestyle causes of chronic disease, which involved individuals taking more responsibility for their own health. But if the public could not be persuaded, where did that leave prevention? The ‘red book’ had sold well, earning a fourth reprint, and 80,000 copies disseminated in 6 months.^96^ Despite this public interest and its status as a consultative document, there was no formal mechanism, however, to involve the public. As the parliamentary inquiry had found, those members of the public that did respond unprompted tended to be on the fringes of mainstream opinion. The government could continue to bang the drum for prevention, but without the participation of the public in either word or action, it was an empty sound. The boundaries of the preventative consensus had been reached.

If there were questions about the means of prevention, there was also scepticism about the methods. A growing disquiet about the merits of health education had been developing as an undercurrent throughout the parliamentary inquiry by witnesses such as the Society of Community Medicine, Yudkin and LSHTM. Owen himself wondered at its merits:

People generally recognise the ill effects of sloth, gluttony and intemperance and would like to be fitter, but they do little about it. Health education to the community and counselling of the individual on many of these subjects are relatively ineffective. To give a concrete illustration, surveys carried out on behalf of the Health Education Council have shown that over 95 per cent of young people are now aware that cigarette smoking causes lung cancer, yet the change in behaviour, implied by this knowledge, has been slight.^97^

This was a strange admission from a minister who had just authorised the publication of *Prevention and Health*, but it does, however, amply illustrate the dichotomy at the heart of this concept of prevention. Individuals’ lifestyles were considered responsible for the proliferation of chronic diseases, but health education was, in many people’s estimation, more or less futile.

Despite this growing atmosphere of scepticism, the command paper itself sought to reframe the debate around health education, with the accompanying press release self-reflexively stating that ‘too often the message … is “don’t”’.^98^ This represented a subtle shift away from the ‘red book’. A more permissive narrative was provided, highlighting the positive aspects of a healthy lifestyle. Rather than the government telling the individual that they should change their behaviour, it reimagined this personal responsibility as ‘positive steps for healthy living’.^99^ But it had little new to report in policy terms, a mere 18 months down the line. Instead, it trailed forthcoming booklets in the *Prevention and Health* series, on pregnancy and childbirth, occupational health services and diet. Most contentiously, it rejected the inquiry’s recommendations on reallocating more funding to prevention.^100^

The White paper received a poor hearing in the press. Ennals had privately expressed anxieties that the document was ‘too vague on food, diet and exercise’ and that it should ‘have something stronger to say’ on smoking.^101^ Those fears were realised by the *BMJ*, which lambasted the document as ‘chicken-hearted’. ‘For the last two years’, it complained that

the DHSS has been singing the praises of a preventive approach to health, and the stream of exhortations, warnings, and advice seems never-ending: yet in terms of positive action the Government has done virtually nothing.^102^

Even the Journal of the Royal Society of Health, a much gentler periodical, was no kinder. ‘It is tempting to apply … the schoolmaster’s comment “This boy thinks. When will he act?”’ it mused, before concluding that a ‘bolder approach to prevention would have been appreciated’.^103^

In the DHSS’ attempts to communicate and publicise their preventative agenda, the limits of the consensus were exposed. Without the participation of the public, and with growing concern about the efficacy of health education, prevention was indeed in danger of becoming little more than a ‘publicity exercise’.

## Conclusion

Prevention as a paradigm was developed in the early 1970s, as a response to epidemiological studies that had highlighted smoking, diet and physical inactivity as risk factors for chronic disease, especially heart disease. This reaction was catalysed by the financial pressures the health service was experiencing, the 1974 reorganisation of the NHS and international awareness of the Lalonde report. Such widespread interest resulted in three different but contemporaneous reports on prevention in 1976 and 1977. All three emphasised, to varying degrees, personal responsibility and lifestyle as important tenets of prevention. This preventative consensus coalesced with shifting ideas about the welfare state and its relationship to the role of the individual. A healthy lifestyle was seen as a citizenly duty on which the continuation of a health service free at the point of use might well depend.

This consensus on prevention can also be viewed in the context of an emerging revisionist historiography of the 1970s which has argued that popular individualism was an important precondition for the Thatcher years, rather than the result of her premiership.^104^  *Prevention and Health* demonstrates that ‘the cult of individual responsibility’ did not start with Thatcher or the New Right.^105^ Rather, Owen and the DHSS had attempted to embed an individualised, lifestyle approach to public health policy that was sustained by the succeeding Conservative administration. Indeed, like that Tory government’s deprecation of the Black report’s findings on publication, *Prevention and Health* skirted contemporary discussions about the inequalities in health, which had resulted in its commissioning in 1977.^106^ Follow-up booklets to *Prevention and Health*, on topics such as diet, alcohol and avoiding heart attacks, continued into the early years of the 1980s.^107^ In highlighting personal responsibility, and later, the slightly shifted focus to the positive aspects of a healthy lifestyle, the prevention agenda articulated by *Prevention and Health* can potentially be seen as governmental attempts, albeit clumsy, to harness popular individualism. Furthermore, it reveals that advocates of these preventative strategies viewed the public in such terms too: as a group of autonomous individuals, free to change their lifestyle regardless of structural factors.

However, the limitations of such an approach were rapidly exposed. Without any formal mechanism to engage the public, *Prevention and Health* was arguably no more than a publicity exercise. By the end of the decade, Owen’s ‘basic rethink’ had curdled into follow-up booklets offering diffident advice. An unwillingness to reallocate resources from conventional, curative medicine meant that the preventative agenda was long on words and short on action. Health education, as the proposed means of communicating prevention to the public, was subject to increasing scrutiny, and in some critics’ eyes, already found to be wanting. The limits of the preventative consensus had been found with the public. ‘Everybody’s business’, to paraphrase one participant at the *Prevention and Health* symposium, all too easily became ‘nobody’s business’.^108^

## Footnotes

1 Department of Health and Social Security (DHSS), *Prevention and Health: Everybody’s Business: A Reassessment of Public and Personal Health* (London: Her Majesty’s Stationary Office (HMSO), 1976), 7, 31.

2
*Ibid*., 38.

3 Royal College of Physicians and British Cardiac Society, ‘Prevention of Coronary Heart Disease. Report of a Joint Working Party of the Royal College of Physicians of London and the British Cardiac Society’, *Journal of the Royal College of Physicians London*, 1976, 10, 1–18; Expenditure Committee, *First Report from the Expenditure Committee together with the Minutes of Evidence taken before the Social Services and Employment Sub-Committee in Sessions 1975–76 and 1976–77 Appendices and Index. Preventive Medicine. Volume I. Report*, 1977.

4 DHSS, *Prevention and Health. Command Paper 7047* (London: HMSO, 1977)

5 Virginia Berridge, ‘Post-war Smoking Policy in the UK and the Redefinition of Public Health’, *Twentieth Century British History*, 2003, 14, 73.

6 Charles Webster, *The National Health Service: A Political History*, 2nd edn (Oxford: Oxford University Press, 2002), 137.

7 Geoff Eley, ‘Historicizing the Global, Politicizing Capital: Giving the Present a Name’, *History Workshop Journal*, 2007, 63, 166. For more context on the economic circumstances in which British policymakers were operating, see Richard Coopey and Nicholas Woodward, ‘The British Economy in the 1970s: An Overview’, in Richard Coopey and Nicholas Woodward, eds, *Britain in the 1970s: The Troubled Economy* (London: UCL Press, 1996), 1–33. The historiography of the 1970s has (broadly) posited the decade in terms of the International Monetary Fund crisis of late 1976, the lead-up to the so-called Winter of Discontent of 1979, and the apparent popular rejection of social democracy that the election of Margaret Thatcher represented. This orthodoxy is represented in academic and popular histories by Alwyn Turner, *Crisis? What Crisis? Britain in the 1970s* (London: Aurum, 2008); Dominic Sandbrook, *Seasons in the Sun: The Battle for Britain 1974–79* (London: Allen Lane, 2012). More nuanced accounts include Joe Moran, ‘“Stand Up and Be Counted”: Hughie Green, the 1970s and Popular Memory’, *History Workshop Journal*, 2010, 70, 172–98; Andy Beckett, *When the Lights Went Out: Britain in the Seventies* (London: Faber, 2009). For this context specifically related to the NHS, see Rudolf Klein, *The New Politics of the NHS: From Creation to Reinvention*, 5th edn (Oxford: Radcliffe, 2006), 77–80; and Webster, *Political History*, 72–74.

8 Neville Hodgkinson, ‘Change in Attitude to NHS Sought’, *The Times*, 9 February 1976, 59623,1.

9 Matthew Grant, ‘Historicising Citizenship in Post-War Britain’, *The Historical Journal*, 2016, 59, 1187–206.

10 Emily Robinson *et al.*, ‘Telling Stories about Post-war Britain: Popular Individualism and the “Crisis” of the 1970s’, *Twentieth Century British History*, 2017, 28, 268–304.

11
*Ibid*., 272. The phrase ‘marketplace of ideas’ is taken from the political economist Peter A. Hall who used it to describe the options open to British economic policymakers in the mid-to-late 1970s. Peter A. Hall, ‘Policy Paradigms, Social Learning, and the State: The Case of Economic Policymaking in Britain’, *Comparative Politics*, 1993, 25, 275–96.

12 DHSS, *Prevention and Health*, 96.

13 Robinson *et al.*, ‘Telling Stories’, 303–04.

14 Grant, ‘Historicising Citizenship’, 1204.

15 Philip Begley *et al.*, eds, *The 1974 NHS Reorganisation: The Transcript of a Witness Seminar*, held 9 November 2016 at the University of Liverpool in London, published by the Department of Public Health and Policy, University of Liverpool, 2017, https://www.liverpool.ac.uk/media/livacuk/iphs/researchgroups/governanceofhealth/PDF1,-,1974_.pdf, accessed 11 February 2019; James Burke, ‘Something for Nothing: A Birthday Celebration’, BBC 1, 27 June 1968, http://www.bbc.co.uk/archive/nhs/5157.shtml, last accessed 2 August 2018. See also discussion of this programme and its handling of race in Roberta Bivins, ‘Picturing Race in the British National Health Service, 1948–1988’, *Twentieth Century British History*, 2017, 28, 107–08.

16 Godber had been CMO since 1960 and had lent significant support to efforts to highlight the health risks of smoking, such as his role in the Royal College of Physicians’ highly influential report on the issue in 1962. For more on Godber, see Sally Sheard and Liam Donaldson, *The Nation's Doctor: The Role of the Chief Medical Officer 1855–1998* (Oxford: Radcliffe, 2005), 201–04. For more on the report and its legacy, see Virginia Berridge, ‘Medicine and the Public: The 1962 Report of the Royal College of Physicians and the New Public Health’, *Bulletin of the History of Medicine*, 2007, 81, 286–311.

17 George Godber, ‘Preventive Medicine in the ’70s’, *Journal of the Royal Society of Health*, 1971, 91, 171.

18 George Newman, *The Rise of Preventive Medicine* (Oxford: Oxford University Press, 1932). For a discussion of Newman’s book and attitude towards prevention, see Sheard and Donaldson, *The Nation's Doctor*, 93–94. John Welshman, ‘Bringing Beauty and Brightness to the Back Streets’: Health Education and Public Health in England and Wales, 1890–1940’, *Health Education Journal*, 1997, 56, 202. See also Max Blythe, *History of the Central Council for Health Education, 1927–1968* (unpublished DPhil, University of Oxford, Green College, 1987).

19 Alex Mold, ‘*“*Everybody Likes a Drink. Nobody Likes a Drunk”. Alcohol, Health Education and the Public in 1970s Britain’, *Social History of Medicine*, 2017, 30, 617; Central Health Services Council and Scottish Health Services Council, *Health Education* (London: HMSO, 1964); Ian Sutherland, *Health Education—Half a Policy, 1968–86: Rise and Fall of the Health Education Council* (Cambridge: NEC Publications, 1987).

20 Dorothy Porter, ‘From Social Structure to Social Behaviour in Britain after the Second World War’, *Contemporary British History*, 2002, 16, 58–80.

21 Jeremiah N. Morris, ‘Four Cheers for Prevention’, *Proceedings of the Royal Society of Medicine*, 1973, 66, 225–32.

22
*Ibid*., 229.

23 Tom W. Meade, ‘Our Lives and Hard Times’, *The Lancet*, 1975, 2, 1053–57; Archibald L. Cochrane, *Effectiveness and Efficiency: Random Reflections on Health Services* (London: Nuffield Provincials Hospital Trust, 1972).

24 Klein, *New Politics*, 79.

25 Office of Health Economics, *OHE Briefing: The Cost of the NHS*, October 1978, https://www.ohe.org/publications/cost-nhs, last accessed 19 November 2018.

26 Trevor Weston, *How is the Health Service?* (London: Conservative Political Centre, 1972).

27 Marc Lalonde, *A New Perspective on the Health of Canadians* (Ottawa: Government of Canada, 1974). For an account of the gestation of the Lalonde report which suggests that it emerged without consultation and quite independently from global policymaking forums such as the World Health Organization, see Hubert Laframboise, ‘Non-Participative Policy Development: The Genesis of “A New Perspective on the Health of Canadians”’, *Journal of Public Health Policy*, 1990, 11, 316–22.

28
*Ibid*., 33.

29 Heather MacDougall, ‘Reinventing Public Health: A New Perspective on the Health of Canadians and its International Impact’, *Journal of Epidemiology and Community Health*, 2007, 61, 956.

30 Lalonde, *A New Perspective*, 13, quoted from Thomas McKeown, ‘An Interpretation of the Modern Rise in Population in Europe’, *Population Studies*, 1972, 27, 345. See also Simon Szreter, ‘The Importance of Social Intervention in Britain's Mortality Decline c. 1850–1914: A Reinterpretation of the Role of Public Health’, *Social History of Medicine*, 1988, 1, 1–38 and Simon Szreter, ‘Rethinking McKeown: The Relationship Between Public Health and Social Change’, *American Journal of Public Health*, 2002, 92, 722–25.

31 Lalonde, *A New Perspective*, 39.

32
*Ibid*., 66.

33 George Godber, ‘McKeown’s “The Role of Medicine”: Comments from a Former Chief Medical Officer’, *Milbank Memorial Fund Quarterly*, 1977, 55, 373.

34 Theodore R. Marmor and Albert Weale, ‘A New Perspective on Health? Learning from Lalonde’, in Theodore R. Marmor and Rudolf Klein, eds, *Politics, Health, and Health Care: Selected Essays* (New Haven: Yale, 2012), 510–11.

35 Robert Evans, ‘A Retrospective on the “New Perspective”’, *Journal of Health Politics, Policy and Law*, 1982, 7, 325–44; Marmor and Weale, ‘A New Perspective’, 511.

36 Anon, ‘Towards Prevention’, *The Lancet*, 1975, 305, 318.

37 John Reid, Letter to John Brotherston, 7 January 1975, The National Archives (TNA), MH 150/829; Webster characterises Owen’s tenure at DHSS as being ‘both energetic and innovative’, while noting that he ‘was only the third medically qualified politician to hold a senior ministerial office in the health department since the formation of the Ministry of Health in 1919’, Webster, *Political History*, 67.

38 Labour Party, *Labour Party Manifesto: October 1974* (London: Labour Party, 1974), 15.

39 J. J. A. Reid, Letter to T. Baird, 10 March 1975, TNA, MH 150/829.

40 A. D. Johnson and A. Yarrow, ‘Minutes of First Meeting of the Steering Committee for the Consultative Paper on Preventive Medicine, 25 March 1975’, TNA, MH 150/829.

41
*Ibid*.

42 Richard Turner and Keith Ball, ‘Prevention of Coronary Heart-Disease: A Counterblast to Present Inactivity’, *The Lancet*, 1973, 302, 1137–40.

43
*Ibid*., 1140.

44 Illana Löwy, *A Woman’s Disease: The History of Cervical Cancer* (Oxford: Oxford University Press, 2011), 107–28; J. M. G. Wilson and G. Jungner, ‘Principles and Practice of Screening for Disease’, *WHO Chronicl*e, 1968, 22, 473.

45 Turner and Ball, ‘Prevention of Coronary Heart-Disease’, 1139–40.

46 Anon, ‘Can I Avoid a Heart Attack?’ *The Lancet*, 1974, 303, 607.

47 Philosopher of science and technology Bruno Latour has described how the ‘technical aspects of science’ are subject to ‘black-boxing’. The methods by which scientific knowledge is constructed are no longer visible once the the ‘fact’ has been established. Bruno Latour, *Science in Action: How to Follow Scientists and Engineers Through Society* (Cambridge, MA: Harvard University Press, 1987), 21. For a critical discussion of risk factor epidemiology, see William G. Rothstein, *Public Health and the Risk Factor: A History of an Uneven Medical Revolution* (Rochester, NY: University of Rochester Press, 2013).

48 Anon, ‘Can I Avoid a Heart Attack?’, 607.

49 G. Ford, Letter to J. J. A. Reid, 10 March 1975, TNA, MH 150/829.

50 G. Wynne Griffith, ‘Outline: Draft Consultative Document on Preventive Medicine’, 27 January 1975, TNA, MH 150/829.

51 E. E. Simpson, Letter to J. J. A. Reid, 4 March 1975, TNA, MH 150/829.

52 F. D. Beddard, Memorandum to J. J. A. Reid, 1 July 1974, TNA, MH 150/829.

53 G. W. Griffith, ‘Outline: Draft Consultative Document on Preventive Medicine’, 27 January 1975, TNA, MH 150/829.

54 C. L. Bourton, Letter to J. J. A Reid, 14 March 1975, TNA, MH 150/829.

55 J. J. A. Reid, Memorandum to H. Yellowlees and P. Rogers, 18 October 1974, TNA, MH 150/829.

56 J. J. A. Reid, ‘Preventive Medicine’, 17 July 1974, TNA, MH 150/829. For a contemporaneous assessment of CHCs, see Rudolf Klein and Jane Lewis, *The Politics of Consumer Representation: A Study of Community Health Councils* (London: Centre for Studies in Social Policy, 1976). For a historical assessment, see Alex Mold, *Making the Patient-Consumer: Patient Organisations and Health Consumerism* (Manchester: Manchester University Press, 2015), 42–68.

57 J. J. A. Reid, Memorandum to H. Yellowlees and P. Rogers, 18 October 1974, TNA, MH 150/829.

58 D. H. D. Burbridge, Letter to J. J. A. Reid, 4 March 1975, TNA, MH 150/829; T. Geffen, Letter to J. J. A. Reid, 3 March 1975, TNA, MH 150/829.

59 F. D. Beddard, Letter to J. J. A. Reid, 3 March 1975, TNA, MH 150/829.

60 Written Answers (Commons): Social Services ‘Preventive Work’, HC Deb 30 June 1975, vol 894 cc277-8W.

61 Commons Sitting: Social Services “Preventive Medicine”, HC Deb 05 August 1975, vol 897 cc215-6.

62 The BMA wrote to the Secretariat of the Subcommittee asking ‘In view of the conflict of time-tabling, could you please tell me the relationship of the document by Dr. Owen to the work of your Sub-Committee … Are these two separate and unrelated exercises, or have they a connection?’ A. J. G. Dickens, Letter to A. Milner-Barry, Parliamentary Archives (PA), HC/CP/5017.

63 Published in 1970, Toffler’s international best-seller made the argument that in the rapid technological advancement of ‘developed’ post-industrial economies, people were left disorientated and disconnected from the society around them. Alvin Toffler, *Future Shock* (London: Bodley Head, 1970).

64 Expenditure Committee *First Report from the Expenditure Committee together with the Minutes of Evidence taken before the Social Services and Employment Subcommittee in Sessions 1975–76 and 1976–77*, *Appendices and Index. Preventive Medicine. Volume III. Minutes of Evidence 5 May–15 July 1976, Appendices and Index*, 1977, 581.

65
*Ibid*., 748. The Society represented those doctors that had previously been Medical Officers of Health prior to the 1974 reorganisation but was distinct from the Faculty of Community Medicine which represented all public health doctors. See M. D. Warren, ‘The Creation of the Faculty of Community Medicine (now the Faculty of Public Health Medicine) of the Royal Colleges of Physicians of the United Kingdom’, *Journal of Public Health Medicine*, 1997, 19, 93–105. For more detail on the changes to bureaucratic structures within public health, see Jane Lewis, *What Price Community Medicine?: Philosophy, Practice and Politics of Public Health Since 1919* (Brighton: Wheatsheaf Books, 1986).

66
*Ibid*., 755.

67 Expenditure Committee, *First report from the Expenditure Committee together with the minutes of evidence taken before the Social Services and Employment Subcommittee in Sessions 1975-76 and 1976–77, Appendices and Index. Preventive Medicine. Volume II. Minutes of Evidence 10 December 1975–28 April 1976*, 1977, 336.

68
*Ibid*., 473–75.

69
*Ibid*., 505.

70 Expenditure Committee, *Volume III*, 580.

71
*Ibid*., 805.

72 G. I. Grant, Letter to A. Milner-Barry, n.d. (probably February 1976), PA, HC/CP/5079.

73 National Dairy Council, ‘The Controversy regarding the Relationship between Fats and Heart Disease’, 1976, PA, HC/CP/5059.

74 T. K. H. Priestnall, Letter to A. Milner-Barry, 4 January 1977, PA, HC/CP/5092.

75 A. Yarrow, Letter to G.W. Griffiths, 30 April 1975, TNA, MH 150/829.

76 Anon, ‘Note of a Meeting with Minister of State (Health) on 9 July 1975 to discuss the Consultative Document on Preventive Medicine’, TNA, MH 150/830.

77 A. D. Bacon, Letter to Hodgetts, 4 November 1974, TNA, MH 148/691.

78 A. Yarrow and E. L. Mayston, ‘Consultative Document on Prevention—Distribution Arrangements’, 5 December 1975, TNA, MH 154/986.

79 R. Myers, Hand-written Note, 3 August 1976, TNA, MH 148/1166.

80 DHSS, *Prevention and Health*, 31.

81
*Ibid*., 92–93.

82
*Ibid*., 95.

83 N. M. Hale, ‘Draft of Consultative Document on Preventive Medicine’, 17 November 1975, TNA, MH 154/986.

84 David Owen, *In Sickness and In Health: The Politics of Medicine* (London: Quartet Books, 1976), 114–15.

85 Anon, ‘Observations on the General Comments’, n.d. probably October 1975, TNA, MH 154/987

86 RCP and BCS, ‘Prevention of Coronary Heart Disease’, 2.

87
*Ibid*., 4.

88 Neville Hodgkinson, ‘Ministry Circulates Guide on Avoiding Heart Disease’, *The Times*, 8 April 1976, 59674,1. Throughout the production of *Prevention and Health*, there had been an ongoing dialogue between the BCS/RCP working party and DHSS officials. For example, G. Ford, Letter to A. Yarrow, 15 July 1975, TNA, MH 150/830.

89 DHSS, *Prevention and Health: Everybody’s Business. Symposium on ‘Involvement in Prevention’, 22 July 1976, Imperial College, London. Report of Proceedings* (London: HMSO, 1977), 10.

90 Anon, ‘Strategy on Prevention: Note for Secretary of State’, n.d., probably February 1976, TNA, MH 148/691.

91 DHSS, *Symposium*, 7.

92 Anon, ‘Prevention Priorities’, *BMJ*, 1977,1, 989.

93 Martin Gorsky and Gareth Millward, ‘Resource Allocation for Equity in the British National Health Service, 1948–89: An Advocacy Coalition Analysis of the RAWP’, *Journal of Health Politics, Policy and Law*, 2018, 43, 69–108.

94 Expenditure Committee, *Volume I*, 304–09.

95 E. L. Marston, Letter to Cornish, 6 February 1977, TNA, MH 148/691.

96 Diana Geddes, ‘£600,000 for Family Planning’, *The Times*, 20 October 1976, 59839:4.

97 Owen, *In Sickness and In Health*, 105.

98 Anon, ‘White Paper on “Prevention and Health”: Government Re-commitment to Priority for Prevention’, 15 December 1977, TNA, BS 6/2828.

99 DHSS, *Command Paper 7047*, 39.

100
*Ibid*., 76–80.

101 M. Moodie, Memo to Benner, 3 October 1977, TNA, MH 148/691.

102 Anon, ‘Targets for Prevention’, *BMJ*, 1977, 1, 1621.

103 Anon, ‘Prevention and Health’, *Royal Society of Health Journal*, 1978, 98, 1.

104 Robinson *et al.*, ‘Telling Stories’, 268–304. See also Lawrence Black and Hugh Pemberton ‘The Benighted Decade? Reassessing the 1970s’, 1–24, in Lawrence Black, Hugh Pemberton and Pat Thane, eds, *Reassessing 1970s Britain* (Manchester: Manchester University Press, 2013).

105 See citing and discussion of sociologist Harriet Bradley in Robinson *et al*., ‘Telling Stories’, 274.

106 Virginia Berridge ‘The Origin of the Black Report: A Conversation with Richard Wilkinson’, *Contemporary British History*, 2002, 16, 120–22.

107 DHSS, *Prevention and Health: Eating for Health* (London: HMSO, 1978); DHSS, *Prevention and Health: Drinking Sensibly* (London: HMSO, 1981); DHSS, *Prevention and Health: Avoiding Heart Attacks* (London: HMSO, 1981). For a discussion of *Drinking Sensibly*, see Mold, ‘*“*Everybody Likes A Drink”’, 631–34.

108 DHSS, *Symposium*, 16.

## Notes

Peder Clark is a Research Assistant in the Centre for History in Public Health at the London School of Hygiene and Tropical Medicine.

